# The uneasy alliance of assessment and feedback

**DOI:** 10.1007/s40037-016-0300-6

**Published:** 2016-09-15

**Authors:** Christopher Watling

**Affiliations:** Office of Postgraduate Medical Education, Schulich School of Medicine and Dentistry Western University, N6A 5C1 London, Ontario Canada

Assessment and feedback can make strange bedfellows. Their goals sometimes lie at cross purposes. Summative assessment, for example, is judgmental, telling us whether or not learners have reached a necessary standard; as Konopasek observes, “when practicing summative assessment, we are acting far more as regulators than educators” [1]. Feedback, on the other hand, is developmental, facilitating learners’ progress. Reconciling judgement and development is a tenuous balancing act.

Published criteria for good assessment suggest that learners should participate in receiving and acting on feedback as part of assessment processes, particularly when the intent of those processes is formative [2]. Feedback is critical to what Norcini calls the ‘catalytic effect’ of formative assessment – its capacity to drive learning forward [2]. And even summative assessment assessments contain a treasure trove of information about learners – information that could be harnessed to guide their future learning. Exactly how we can encourage learners to meaningfully engage with the feedback embedded in these assessments is a vexing challenge for educators.

Harrison and colleagues grapple with this problem in their exploration of students’ receptivity to formative feedback across three different medical schools, in three different countries, with three distinct approaches to assessment [3]. Their refreshing approach considers the influence of institutional assessment culture on learner behaviour. Recent calls for medical schools to establish cultures that emphasise learning over performance [1] – cultures where coaching for improvement supplants punishment for failure [4] – compel us to understand how such cultures are constituted.

Harrison’s work is part of a growing trend in medical education research toward exploring issues from a sociocultural point of view. Sociocultural learning theories offer a perspective that positions culture, context, system, and organization at the forefront of learning. They direct researchers to examine the big picture, looking not only at the minds of individual learners, but also at the professional communities those learners are joining, and at the institutional environments within which those learners are developing [5]. Pedagogical approaches, including assessment strategies, are not accidental; rather, they reflect a series of deliberate educational choices – some principled, some pragmatic. And the educational choices made by a discipline or profession, in turn, mirror its values [6]. In my view, any exploration of an assessment culture cannot stop at a description of its programs and practices. We should dig deeper, unearthing the values those practices represent and sustain, and the influence of those values on learners’ behaviour. We should ask whether the values our assessment culture reflects are the values we want our future doctors to embrace.

Harrison offers a ‘focused lens’ on the interactions between learners and their contexts, showing that assessment cultures that afford greater student choice and independence reap the reward of greater student feedback receptivity [3]. This finding is novel, but should not surprise us. Medicine’s professional culture values independence and autonomy [7]. Learners aspire to gradually shed their need for supervision and guidance. Assessment strategies that align with these core professional values may therefore be more likely to encourage desirable learner behaviour. When learners can see assessment as enabling their independence, they may buy in more readily.

Harrison further suggests that students ignore feedback they deem to lack credibility [3], reinforcing earlier research on the central place of credibility in the interpretation and use of feedback [8]. Credibility itself, however, is culturally and contextually defined [9]. Credibility in medicine tends to link to authentic clinical work – excellent clinicians are credible sources of feedback, and real cases are credible venues. Harrison cautions that a summative assessment culture may distort notions of credibility for learners. The finding that learners often ignored feedback from real clinical settings as they prepared for OSCEs – perceiving that it was not credible in the context of producing the performance required to pass the exam [3] – provides a disconcerting glimpse into the unintended influence of an assessment culture that fails to align with the profession’s values.

Summative assessment represents a particular challenge for feedback. Tempting as it may be to bemoan the ‘summative assessment culture’ as promoting undesirable, test-focused learning strategies that suffocate professional growth, learners and educators alike recognise the need for summative assessment. Medicine’s culture of summative assessment reflects the profession’s pact with society. The summative assessment culture supports a core professional value – the duty to provide safe, competent care. Illuminating as it may be to look longingly to music or sports as cultures in which coaching can thrive, medicine’s social accountability mandate changes the conversation. Sports coaches and music teachers are accountable to their students. Medical teachers are accountable to their students, but also to their patients and to their communities. While summative assessment has likely been over-emphasised in medicine’s learning culture, it cannot be lifted out completely. But medicine’s reliance on summative assessment has consequences for feedback. Students in Harrison’s study did not seek feedback after summative assessment, reinforcing his earlier work demonstrating that the uptake and use of feedback after assessment was undermined when the assessment was summative [3, 10]. The problem is not with summative assessment itself, but with summative assessment as the dominant learning culture for students.

Perhaps it is expecting too much for truly summative assessment to double as a consistent generator of meaningful feedback. But students often perceive assessment as summative, even when it is not intended that way. For a program of assessment to meet its potential to shape learning, all the players need to understand ‘what they are doing, why they are doing it, and why they are doing it this way’ [11]. Assessment intended as formative needs to be clearly understood by all players as developmental and learning-focused. And perhaps formative assessment, at times, needs to be ‘no-stakes’ as opposed to ‘low-stakes,’ in order to create safe opportunities for coaching within which learners can expose their weaknesses without fear of consequences.

Harrison and colleagues conclude by calling for the gradual development of a culture of receptivity to feedback [3]– a task of diabolical difficulty. Where they offer fresh hope that this goal may be achievable is in their turn away from the usual suspects – training teachers to be better givers of feedback and students to be better receivers. Instead, they focus on curricular decisions made at the institutional level, and highlight the downstream effects these decisions have on learners. In thinking less about individuals and more about culture, new opportunities emerge to enable assessment and feedback to take their place as comfortable partners in learning.
